# Hexaamminecobalt(III) hexa­cyanido­manganate(III)

**DOI:** 10.1107/S1600536808032881

**Published:** 2008-10-18

**Authors:** Hendrik G. Visser, Walter Purcell

**Affiliations:** a Department of Chemistry, University of the Free State, PO Box 339, Bloemfontein 9300, South Africa

## Abstract

The asymmetric unit of the title compound, [Co(NH_3_)_6_][Mn(CN)_6_], contains one Co and one Mn atom, both lying on threefold inversion axes, and one NH_3_ and one CN group. The octa­hedral environments around Co^II^ and Mn^II^ are generated by symmetry and show very slight deviations from ideal geometry. A three-dimensional network is created by N—H⋯N hydrogen bonds.

## Related literature

For related structures, see: Buschmann *et al.* (1999 ▶). For the construction of clusters and networks with adjustable magnetic properties, see: Przychodzen *et al.* (2006 ▶); Withers *et al.* (2005 ▶).
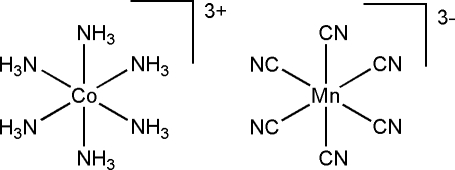


## Experimental

### 

#### Crystal data

[Co(NH_3_)_6_][Mn(CN)_6_]
*M* *_r_* = 372.19
Trigonal, 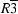

*a* = 10.963 (5) Å
*c* = 10.779 (5) Å
*V* = 1121.9 (9) Å^3^
*Z* = 3
Mo *K*α radiation
μ = 1.96 mm^−1^
*T* = 100 (2) K
0.35 × 0.26 × 0.25 mm

#### Data collection

Bruker SMART APEXII CCD area-detector diffractometer
Absorption correction: multi-scan (*SADABS*; Bruker, 2004 ▶) *T*_min_ = 0.546, *T*_max_ = 0.614
3602 measured reflections
628 independent reflections
497 reflections with *I* > 2σ(*I*)
*R*_int_ = 0.038

#### Refinement

*R*[*F* ^2^ > 2σ(*F* ^2^)] = 0.025
*wR*(*F* ^2^) = 0.071
*S* = 0.93
628 reflections
32 parameters
H-atom parameters constrained
Δρ_max_ = 0.29 e Å^−3^
Δρ_min_ = −0.43 e Å^−3^

### 

Data collection: *APEX2* (Bruker, 2005 ▶); cell refinement: *SAINT-Plus* (Bruker, 2004 ▶); data reduction: *SAINT-Plus* and *XPREP* (Bruker, 2004 ▶); program(s) used to solve structure: *SHELXS97* (Sheldrick, 2008 ▶); program(s) used to refine structure: *SHELXL97* (Sheldrick, 2008 ▶); molecular graphics: *DIAMOND* (Brandenburg & Putz, 2005 ▶); software used to prepare material for publication: *SHELXL97* and *WinGX* (Farrugia, 1999 ▶).

## Supplementary Material

Crystal structure: contains datablocks global, I. DOI: 10.1107/S1600536808032881/pk2122sup1.cif

Structure factors: contains datablocks I. DOI: 10.1107/S1600536808032881/pk2122Isup2.hkl

Additional supplementary materials:  crystallographic information; 3D view; checkCIF report

## Figures and Tables

**Table 1:** Hydrogen-bond geometry (Å, °)

*D*—H⋯*A*	*D*—H	H⋯*A*	*D*⋯*A*	*D*—H⋯*A*
N1—H1*A*⋯N2^i^	0.89	2.09	2.979 (2)	173

Symmetry code: (i) 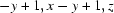.

**Fig. 1.**
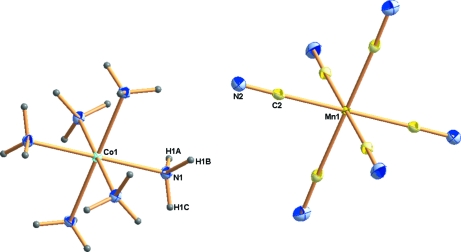
An ellipsoid plot of the title compound (50% probability displacement ellipsoids).

## supplementary crystallographic information

### Comment

Our interest is in the use of cyanometalates as molecular building blocks for potentially constructing clusters and networks with adjustable magnetic properties [Przychodzen *et al.*, (2006), Withers *et al.*, (2005)].

The title compound crystallizes in the trigonal *R*-3 space group with *Z* = 3. The main part of the asymmetric unit contains one Co and one Mn atom, both lying on threefold rotational axes.

The octahedral environments around Co^II^ and Mn^II^ are generated by symmetry and shows very slight deviation from ideal geometry as illustrated by the C—Mn—C angles of 180.00 (7), 89.80 (7) and 90.20 (7) and N—Co—N angles of 180.00 (7), 89.47 (6) and 90.53 (6) ° respectively.

A three dimensional network is created by hydrogen bonds of the type N—H—N.

### Experimental

Equimolar amounts of K_3_[Mn(CN)_6_] and [Co(NH_3_)_6_]Cl_3_ were dissolved in water, added together and allowed to stand. Orange crystals separated out after a few days.

### Crystal data

**Table:**

[Co(NH_3_)_6_][Mn(CN)_6_]	*D*_x_ = 1.653 Mg m^−^^3^
*M**_r_* = 372.19	Mo *K*α radiation, λ = 0.71069 Å
Trigonal, *R*3	Cell parameters from 1532 reflections
Hall symbol: -R 3	θ = 2.9–28.3°
*a* = 10.963 (5) Å	µ = 1.96 mm^−^^1^
*c* = 10.779 (5) Å	*T* = 100 K
*V* = 1121.9 (9) Å^3^	Cuboid, orange
*Z* = 3	0.35 × 0.26 × 0.25 mm
*F*(000) = 570	

### Data collection

**Table:**

Bruker SMART APEXII CCD area-detector diffractometer	497 reflections with *I* > 2σ(*I*)
φ and ω scans	*R*_int_ = 0.038
Absorption correction: multi-scan (SADABS; Bruker, 2004)	θ_max_ = 28.3°, θ_min_ = 2.9°
*T*_min_ = 0.546, *T*_max_ = 0.614	*h* = −12→14
3602 measured reflections	*k* = −14→9
628 independent reflections	*l* = −10→14

### Refinement

**Table:**

Refinement on *F*^2^	0 restraints
Least-squares matrix: full	H-atom parameters constrained
*R*[*F*^2^ > 2σ(*F*^2^)] = 0.025	*w* = 1/[σ^2^(*F*_o_^2^) + (0.0379*P*)^2^ + 3.8029*P*] where *P* = (*F*_o_^2^ + 2*F*_c_^2^)/3
*wR*(*F*^2^) = 0.071	(Δ/σ)_max_ < 0.001
*S* = 0.93	Δρ_max_ = 0.29 e Å^−^^3^
628 reflections	Δρ_min_ = −0.42 e Å^−^^3^
32 parameters	

### Special details

**Table:**

Geometry. All e.s.d.'s (except the e.s.d. in the dihedral angle between two l.s. planes) are estimated using the full covariance matrix. The cell e.s.d.'s are taken into account individually in the estimation of e.s.d.'s in distances, angles and torsion angles; correlations between e.s.d.'s in cell parameters are only used when they are defined by crystal symmetry. An approximate (isotropic) treatment of cell e.s.d.'s is used for estimating e.s.d.'s involving l.s. planes.

### Fractional atomic coordinates and isotropic or equivalent isotropic displacement parameters (Å^2^)

**Table:**

	*x*	*y*	*z*	*U*_iso_*/*U*_eq_	
Co1	0	0	0	0.00732 (17)	
Mn1	0.3333	0.6667	0.1667	0.00607 (17)	
N1	−0.02748 (15)	0.13048 (15)	−0.10659 (13)	0.0107 (3)	
H1A	0.0238	0.2179	−0.0777	0.016*	
H1B	−0.1181	0.1061	−0.1065	0.016*	
H1C	−0.0006	0.1264	−0.1837	0.016*	
C2	0.31385 (18)	0.80238 (18)	0.06080 (16)	0.0119 (3)	
N2	0.30018 (17)	0.87891 (17)	−0.00283 (15)	0.0184 (4)	

### Atomic displacement parameters (Å^2^)

**Table:**

	*U*^11^	*U*^22^	*U*^33^	*U*^12^	*U*^13^	*U*^23^
Co1	0.0076 (2)	0.0076 (2)	0.0067 (3)	0.00381 (10)	0	0
Mn1	0.0064 (2)	0.0064 (2)	0.0055 (3)	0.00318 (10)	0	0
N1	0.0117 (7)	0.0099 (7)	0.0111 (7)	0.0058 (6)	0.0007 (5)	0.0010 (5)
C2	0.0108 (8)	0.0144 (8)	0.0115 (8)	0.0070 (7)	0.0001 (6)	−0.0026 (6)
N2	0.0200 (8)	0.0248 (9)	0.0159 (7)	0.0152 (7)	0.0014 (6)	0.0013 (6)

### Geometric parameters (Å, °)

**Table:**

Co1—N1^i^	1.9718 (16)	Mn1—C2^vii^	1.9696 (19)
Co1—N1^ii^	1.9718 (16)	Mn1—C2^viii^	1.9696 (19)
Co1—N1^iii^	1.9718 (15)	Mn1—C2^ix^	1.9696 (19)
Co1—N1^iv^	1.9718 (15)	Mn1—C2^x^	1.9696 (19)
Co1—N1	1.9718 (15)	N1—H1A	0.89
Co1—N1^v^	1.9718 (15)	N1—H1B	0.89
Mn1—C2^vi^	1.9696 (19)	N1—H1C	0.89
Mn1—C2	1.9696 (19)	C2—N2	1.150 (2)
			
N1^i^—Co1—N1^ii^	180.00 (7)	C2—Mn1—C2^viii^	89.80 (7)
N1^i^—Co1—N1^iii^	89.47 (6)	C2^vii^—Mn1—C2^viii^	89.80 (7)
N1^ii^—Co1—N1^iii^	90.53 (6)	C2^vi^—Mn1—C2^ix^	89.80 (7)
N1^i^—Co1—N1^iv^	89.47 (6)	C2—Mn1—C2^ix^	90.20 (7)
N1^ii^—Co1—N1^iv^	90.53 (6)	C2^vii^—Mn1—C2^ix^	180
N1^iii^—Co1—N1^iv^	89.47 (6)	C2^viii^—Mn1—C2^ix^	90.20 (7)
N1^i^—Co1—N1	90.53 (6)	C2^vi^—Mn1—C2^x^	89.80 (7)
N1^ii^—Co1—N1	89.47 (6)	C2—Mn1—C2^x^	90.20 (7)
N1^iii^—Co1—N1	180.00 (10)	C2^vii^—Mn1—C2^x^	90.20 (7)
N1^iv^—Co1—N1	90.53 (6)	C2^viii^—Mn1—C2^x^	180.00 (7)
N1^i^—Co1—N1^v^	90.53 (6)	C2^ix^—Mn1—C2^x^	89.80 (7)
N1^ii^—Co1—N1^v^	89.47 (6)	Co1—N1—H1A	109.5
N1^iii^—Co1—N1^v^	90.53 (6)	Co1—N1—H1B	109.5
N1^iv^—Co1—N1^v^	180.00 (10)	H1A—N1—H1B	109.5
N1—Co1—N1^v^	89.47 (6)	Co1—N1—H1C	109.5
C2^vi^—Mn1—C2	180	H1A—N1—H1C	109.5
C2^vi^—Mn1—C2^vii^	90.20 (7)	H1B—N1—H1C	109.5
C2—Mn1—C2^vii^	89.80 (7)	N2—C2—Mn1	178.31 (17)
C2^vi^—Mn1—C2^viii^	90.20 (7)		

Symmetry codes: (i) *y*, −*x*+*y*, −*z*; (ii) −*y*, *x*−*y*, *z*; (iii) −*x*, −*y*, −*z*; (iv) *x*−*y*, *x*, −*z*; (v) −*x*+*y*, −*x*, *z*; (vi) −*x*+2/3, −*y*+4/3, −*z*+1/3; (vii) −*y*+1, *x*−*y*+1, *z*; (viii) −*x*+*y*, −*x*+1, *z*; (ix) *y*−1/3, −*x*+*y*+1/3, −*z*+1/3; (x) *x*−*y*+2/3, *x*+1/3, −*z*+1/3.

### Hydrogen-bond geometry (Å, °)

**Table:**

*D*—H···*A*	*D*—H	H···*A*	*D*···*A*	*D*—H···*A*
N1—H1A···N2^vii^	0.89	2.09	2.979 (2)	173

Symmetry codes: (vii) −*y*+1, *x*−*y*+1, *z*.
